# Experiences of How Health and Lifestyle among Individuals with Knee Pain Have Been Influenced during the COVID-19 Pandemic, a HALLOA Study

**DOI:** 10.3390/ijerph19148255

**Published:** 2022-07-06

**Authors:** Evelina Sunesson, Charlotte Sylwander, Emma Haglund, Maria L. E. Andersson, Ingrid Larsson

**Affiliations:** 1 Spenshult Research and Development Centre, SE-30274 Halmstad, Sweden; evelina.sunesson@fou-spenshult.se (E.S.); charlotte.sylwander@hh.se (C.S.); emma.haglund@hh.se (E.H.); maria.andersson@fou-spenshult.se (M.L.E.A.); 2 Department of Health and Care, School of Health and Welfare, Halmstad University, SE-30118 Halmstad, Sweden; 3 Section of Rheumatology, Department of Clinical Sciences, Lund University, SE-22242 Lund, Sweden; 4 Department of Environmental and Biosciences, School of Business, Innovation and Sustainability, Halmstad University, SE-30118 Halmstad, Sweden

**Keywords:** COVID-19, knee pain, health, lifestyle, adjusting behaviour, revaluing life, qualitative content analysis, interviews

## Abstract

The COVID-19 pandemic has affected the health and lifestyles of both the general population and of vulnerable groups. Individuals with knee pain are recommended to lead an active lifestyle to relieve pain but find it difficult to maintain health and lifestyle compared to the general population due to the cause of chronic pain, impaired physical function, and a diminished quality of life. This study aimed to explore experiences of how health and lifestyle among individuals with knee pain have been influenced during the COVID-19 pandemic. Interviews (n = 19) were conducted in 2021 and analysed with qualitative content analysis. The results showed how individuals with knee pain adjusted their behaviour and revalued their life to maintain health and lifestyle during COVID-19. Adjusted behaviours emerged, such as spending more time at home, becoming digital, and spending more time outdoors, while revaluing life emerged as having a positive outlook on life and sharing responsibility. In conclusion, behaviour was adjusted, and life revalued to manage health and lifestyle during the pandemic. However, the findings are probably similar to the general population, i.e., individuals with knee pain live similar lives as the general population despite knee pain. The results may contribute to alternative ways of maintaining health and lifestyle in various vulnerable groups and may be applied in situations other than the pandemic.

## 1. Introduction

Knee osteoarthritis (KOA) is the most common joint disease, affecting 19–43% of the global population aged 40 and older [1,2]. An early sign of KOA can be knee pain, and results from a population-based cohort study showed that 97% of individuals with knee pain develop KOA over time [3]. KOA can cause chronic pain, impaired physical function, diminished quality of life [4], and affects workability [5]. Individuals with knee pain also have a higher risk of developing chronic widespread pain (CWP) [6], and in a previous study the prevalence of CWP was 38% of those with knee pain [7]. Health is more than the absence of disease; it is a state of physical, mental, and social well-being [8]. Individuals with knee pain find it difficult to maintain health and lifestyle compared to the general population [9]. Information, education, assistive technology and adaptations, lifestyle changes to manage exercise and weight loss, pharmaceuticals, and surgery are treatments to relieve pain and improve health and physical function among individuals with knee pain [10].

The coronavirus disease 2019 (COVID-19) pandemic has affected the lives of nearly every individual around the world [11]. To avoid the rapid spread of the virus, countries adopted restrictions to minimise mortality and morbidity in the population through communicable disease prevention measures such as physical distancing, isolation, and safeguarding essential workers and individuals at risk [12,13,14]. These restrictions have resulted in a radical change of health and lifestyle for the general population and for vulnerable groups; sedentary behaviour, lack of social participation, life dissatisfaction, and unemployment has had a negative effect on health [15,16,17]. The pandemic has contributed to a delay in non-emergency procedures at hospitals, resulting in challenges for patients with knee pain to receive care [18]. This state of uncertainty about an individual’s future, as well as about the future of one’s family and community has been reported to lead to states of frustration, anxiety, fear, and stress [19]. Socializing to avoid feeling lonely and having friends and family who need support have been found to be barriers to adherence to recommendations of physical distancing [20]. Prioritising one’s own and others’ health by respecting the restrictions in place to avoid passing the virus to others have been found to be a facilitator of adherence to physical distancing in society [20]. Experiences of togetherness have been reported in families during the pandemic, but so have a lack of private time and space for oneself [21]. Digital platforms have been used to increase physical activity and to decrease pain and loneliness before and during the pandemic [22,23,24].

Individuals with knee pain find it difficult to maintain health and lifestyle compared to the general population because of chronic pain, impaired physical function, and a diminished quality of life. The pandemic has affected everyday life, health, and lifestyle in the general population, so it is important to explore how to preserve health and lifestyle during the pandemic among vulnerable groups such as individuals with knee pain. The aim was to explore experiences of how health and lifestyle among individuals with knee pain have been influenced during the COVID-19 pandemic.

## 2. Materials and Methods

### 2.1. Design

This qualitative study had an explorative design, in which qualitative content analysis with an inductive approach emphasized variation in experiences [25,26].

### 2.2. Context

Sweden’s response to the COVID-19 pandemic is based on the same pandemic plans as other countries: to minimise mortality and morbidity through communicable disease prevention measures [13]. The Swedish response has implemented restrictions without any full-scale lockdown to minimise negative public health effects and safeguard essential workers. Risk groups were targeted by specific restrictions, particularly the population over 70 years of age. To minimise contacts among individuals, physical distancing was applied and areas of risk in terms of acquiring the virus were identified and restricted. Quarantine, enhanced hygiene routines, and personal protective equipment, in combination with testing regimes and contact tracing, have been additional ways of limiting the spread of the virus [13].

### 2.3. Participants

The study was based on an ongoing longitudinal cohort study (the Halland Knee Osteoarthritis cohort study, HALLOA) that included 306 individuals with knee pain in Southwest Sweden, as described in detail elsewhere [7]. Participants from the HALLOA study who had performed the two-year follow-up after inclusion were eligible for the current study (n = 101). Thirty individuals were invited to participate, of which 19 were included (12 women, 7 men). The purposeful sample consisted of a variety of women and men, ages, civil status, educational levels, body mass index (BMI), radiographic KOA, pain distribution, and activity level. The participants are further described in Table 1 (baseline data).

### 2.4. Data Collection

Individual semi-structured interviews were conducted between February and May 2021. Due to the pandemic, the participants were given the option to perform the interviews by telephone (n = 12), face-to-face using a web-based videoconferencing (n = 5), or in person (n = 2) at a research and development centre. All interviews were initiated with open-ended questions: “Tell me about your health today and how you experience it”, “What enables you to maintain health?”, “What impact have you experienced on your health during the COVID-19 pandemic?”, “What impact have you experienced on your lifestyle during the COVID-19 pandemic?, and “What activities or strategies have you changed to maintain your health during the COVID-19 pandemic?”. To elicit rich data, follow-up questions were asked, such as “Please, can you tell me more about …?”, or “How do you mean?”. A pilot interview was conducted to test the accuracy of the questions. The interviews were performed by the researcher (ES) and (CS) and ranged from 42 to 93 min, with a total interview length of 23 h and 32 min. The interviews were digitally recorded and transcribed verbatim.

### 2.5. Data Analysis

The qualitative content analysis follows the six steps according to Graneheim and Lundman (2004). To gain an overview of the material, the transcribed interviews were listened to and read through several times [25,26]. Text related to the aim was divided into 423 meaning units. Each meaning unit was condensed and abstracted into codes describing the same content. The codes were grouped into five sub-categories based on similarities and differences, which formed two categories. For example, the code socialising digital was grouped into the sub-category becoming digital and sorted to the category adjusting behaviours because of the COVID-19 pandemic (Table 2). The first author (ES) performed the analysis, and the last author (IL) acted as co-assessor. A continual discussion of the analysis occurred between ES and IL, including a movement between the whole and parts of the text, and in which the sub-categories and categories were revised several times. The analysis was frequently discussed with all co-authors to reach consensus.

### 2.6. Ethical Considerations

The study adhered to the Declaration of Helsinki and was approved by the Swedish Ethics Review Authority (No 2016/816; 2017/205; 2020-04489) and registered at ClinicalTrials.gov (NCT04928170) [27,28]. Written and oral information on the study, including the voluntary nature of participation, were given, and the participants signed a written informed consent prior to inclusion in the study. All participants were informed that they could withdraw from the study at any time [27].

## 3. Results

The results revealed that individuals with knee pain experienced adjusted behaviours and revalued life as a result of the COVID-19 pandemic (Table 3).

### 3.1. Adjusting Behaviors Because of the COVID-19 Pandemic

This category was based on the sub-categories of spending time at home, becoming digital, and spending time outdoors. To maintain health and lifestyle was behaviour adjusted among individuals with knee pain because of the COVID-19 pandemic.

#### 3.1.1. Spending Time at Home

When national recommendations called for physical distancing in connection with the COVID-19 pandemic, social restrictions emerged, resulting in more time at home and less physical contact. Participants spoke of not meeting friends and families as usual, and that they had become more reclusive. Spending prolonged periods of time at home was, for some participants, stressful, and some spoke of the occurrence of mental illness and poor health within families. Difficulties in recovery from work were expressed as being a result of spending more time at home than before the pandemic. Some participants found it difficult to recharge because the restrictions meant that they could not go on vacation abroad or have a social life. Conversely, spending time with family members within the household and not being forced to drive to work or to take part in activities were spoken of as relaxing, and also encouraged self-reflection.

*Right now it feels like you should try to keep to yourself a lot. It’s the family that is trying to figure out what they can do now.*

(Participant no. 11)

The national recommendations for physical distancing also hindered the possibility of maintaining an exercise routine to relieve knee pain and manage physical function. Participants spoke of entering long inactive periods when the pandemic emerged as a result of the closures of swimming pools, gyms, and other activities. However, exercising at home was an activity that some participants used to maintain health and lifestyle.

*I have the ambition to get started with some strength training again, but have waited. A bit because of what we find ourselves in today, with the pandemic, but I have started to do a little bit here at home.*

(Participant no. 14)

#### 3.1.2. Becoming Digital

The COVID-19 pandemic was described as enforcing a digital transition among the participants, where they engaged in more digital platforms than before the pandemic. Digital platforms were used to keep in touch with friends, families, and relatives, which facilitated companionship and togetherness when feelings of loneliness and isolation were present. However, socializing digitally was spoken of as not being an adequate substitute for replacing hugs, physical contact, or the sense of connection between human beings.

*It’s really this close contact you get with people. Humans probably need this physical contact in some way as well, and not just sit and watch a screen.*

(Participant no. 22)

For some participants, the longing to meet in person was challenging to cope with when socializing digitally. Likewise, it was challenging to interact digitally with elderly individuals who did not have the technology, or they lacked the digital literacy to engage in digital platforms.

*No, it’s just been a phone call now and again. Grandma only has a normal old-fashioned mobile phone and no data or things like that. There is no FaceTime or anything advanced in that direction. There is mostly just absence, quite simply.*

(Participant no. 16)

Participants spoke of an accelerated digital transition in many workplaces, where work tasks have become more efficient. Those who worked from home described that they had more time left over to engage in household activities and felt more creative, and were inspired to manage work tasks better. However, engaging in video conferencing from home was also considered to reduce creativity among some participants, and building relationships with colleagues or customers was difficult for some. Participants mentioned that adaptive behaviours were important to deal with the changes involved in managing their work digitally.

*I can imagine that if I had had physical meetings, then maybe something different would have happened, but I don’t really know today what it could be. So that’s what I mean, that creativity and that ideas thinking and that flow I don’t think work in the same way as when you meet. For various reasons, I think. Maybe you also have more respect for the fact that a person has travelled a long way. You may be more prepared than these meetings. Yes, okay now there’s a new meeting at 10, and there’s one at 1 pm and then it’s a little more casual and you get it over with in a different way. While you have a different respect for a physical encounter, I can imagine.*

(Participant no. 14)

Digital platforms were also used to continue exercising, engage in rehabilitation, or maintain health and lifestyle during the pandemic. Lack of previous exercise experience was a barrier to engaging in digital activities, making it difficult to maintain exercise for long. Online shopping was spoken of as an activity to save time and avoid physical contact. However, the ability to support local stores and the sensation to stroll in a store were mentioned as meaningful and important to consider when proceeding with the digital transition to online shopping.

*You are surprisingly adaptable too, I think. Especially at work, that all of a sudden everyone was at home and sat at home and worked. All this with digitization and so on, that it went so fast that you adapt to it.*

(Participant no. 18)

#### 3.1.3. Spending Time Outdoors

Outdoor activities became a way of socializing with friends and family members and to cope with physical distancing. Participants described being safer meeting outside because the virus could not spread as easily outdoors as indoors. Meeting outdoors was easy to arrange, with less preparation than inviting friends home for dinner, and hiking, walks, and cooking over an open fire were popular activities. Cold or rainy weather was mentioned as hindering outdoor activities for some participants. In contrast, others spoke of being solution orientated and finding a way of meeting or arranging outdoor activities despite the weather. Some said that the pandemic had influenced their lifestyle in that they spent more time outdoors than before the pandemic, while others spoke of not engaging in outdoor activities.

*I understand my parents a bit, that they miss meeting and so on. Mum has talked a bit that “now it’s your turn to come home and eat, when the weather is just a little better so we can sit outside”.*

(Participant no. 22)

Due to closures of swimming pools, gyms, and other activities, outdoor activities such as walking, golf, and biking became a way of maintaining health and lifestyle. For one family, the increased outdoor activity brought about by the pandemic was the reason that they got a dog. Walks or cycling outings often replaced the use of public transportation during the pandemic, which changed the participants’ lifestyles and was viewed as good for the health.

*It doesn’t have to be that you hang out with your friends on a Saturday night, that you should invite each other home or whatever it is that you do. Without being able to ring each other one day in the middle of the week after work. “Shall we go for a walk this evening?”. So you meet and walk and talk at the same time. It’s a nice way to hang out too.*

(Participant no. 6)

### 3.2. Revaluing Life Because of the COVID-19 Pandemic

This category was based on the sub-categories of having a positive outlook on life and sharing responsibility. These aimed to describe how revaluing and appreciating life became important to maintain health and lifestyle during the COVID-19 pandemic among individuals with knee pain.

#### 3.2.1. Having a Positive Outlook on Life

Although the COVID-19 pandemic brought fear and uncertainty about the future, having a positive outlook on life helped them to cope with the pandemic. When events, trips, family gatherings, and non-emergency surgeries to relieve knee pain were cancelled or postponed, it was considered important to have a positive attitude towards life. Some emphasised the importance of maintaining health and lifestyle despite the pandemic and being solution-oriented to find the best possible outcomes so that they could continue to live positively.

*It’s hard of course, because it’s corona. But I can’t, I can’t just bury myself. You still have to have common sense. The guidelines, of course, you have to keep them all, but I don’t have to stop living because of it.*

(Participant no. 4)

Participants said that they had begun to place more value on the little things in life and focus on what made them feel grateful. Among the topics that arose were thankfulness that life carried on, for spending more time with close family members and knowing they are doing well, for having a job, for moving around freely outside and not being restricted to staying only at home. As an outcome of complying with physical distancing recommendations, participants spoke of being healthier than before the pandemic, with fewer viral infections. Not being expected or forced to meet relatives or friends on weekends and for holidays was sometimes viewed as a relief. Some participants spoke of beginning to set boundaries to maintain their health, with fewer gatherings with friends and family.

*I don’t have a huge amount of social interaction in the first place. I haven’t been hit so hard by not being able to meet other people and socialize. Then there are certain social things that usually feel awkward and that I have to do, even though really I don’t want to. Now I have been able to blame the fact that it’s not possible, now when it’s Corona. So, we have ignored all that stuff and got rid of that hard boring stuff. In that way, it has been really positive. I have been able to blame Corona, although really I don’t want to.*

(Participant no. 16)

#### 3.2.2. Sharing Responsibility

The participants expressed that being responsible for others facilitated compliance with the rules of maintaining physical distance during the COVID-19 pandemic. To reduce the risk of spreading infection, keeping distance from others and choosing to go food shopping alone or attending the gym when it was less crowded were spoken of as necessary, as was reminding oneself to comply with the restrictions to show consideration and respect towards oneself and others. Some mentioned that our behaviour reflects upon others, which could help us remember to comply with the restrictions.

*You have to set up your own rules in some way. I think that one has adapted. I really think you notice it, that if you are somewhere in a shop or something that you do, people go a little to the side and so on. Then you don’t have to expose yourself to that type of* *thing, where you are in situations where it’s very crowded and things like that.*

(Participant no. 18)

Finding ways to support elderly individuals in maintaining everyday life routines was considered important, such as by purchasing food and medications and leaving it outside the door and arranging family gatherings with distance from each other to reduce the risk of spreading infection. Staying in isolation and not meeting others was considered important before driving an elderly family member to a health appointment, which also meant the avoidance public transport or taxis.

*I’m actually trying to do it my way. I took the COVID test when I was going to drive my father, who was going to go and have a heart valve changed. Just to check that I didn’t have it.*

(Participant no. 12)

Feelings of helplessness and sadness arose when restrictions hindered the ability to support the elderly. Not being able to visit an older person at a retirement home who could not keep in touch by phone or digital platforms was spoken of as difficult; it also created new perspectives, where life was viewed as fragile. Participants mentioned that if someone close to them was suffering, it led them to revalue life and shift the focus from oneself to one’s loved one. Some participants spoke of choosing not to see elderly family members because of the hard fact that they could not hug or touch them. Meeting with distance was mentioned as challenging and even more difficult for the younger ones in the families.

*It was a little hard before because my mother-in-law was ill. She’s dead now. But she was sad because I was up with her a lot and helped her before the pandemic. But when it came, they got it. This Corona. So there were such restrictions so it was dead hard. Yes, I tried anyway, you know. When I was out shopping I bought a plant and I gave it to the staff so that it would give it to her. So that she would still feel that we are out here for her anyway. But she decided she did not want to live anymore because she thought it was so hard. I was the one who took care of her, my mother-in-law. So now that it was like this, I couldn’t just come in and have coffee. She loved that I did. It wasn’t possible now.*

(Participant no. 15)

## 4. Discussion

This study highlights how individuals with knee pain adjusted their behaviour and revalued life to maintain health and lifestyles during COVID-19. Adjusted behaviours emerged, such as spending more time at home, becoming more digital, and spending more time outdoors, while revaluing life emerged as having a positive outlook on life and sharing responsibility.

Spending more prolonged periods of time at home was found to be a consequence of the COVID-19 pandemic which impacted health in the present study. More reclusive behaviour had led to fewer social interactions outside the household, and strained family relations. Conversely, spending more time at home was also found, to improve health behaviour in the present study by strengthening family relationships. These results align with those of Evans et al. (2020), who explored the impact of the pandemic among Australian families. Strengthened relationships were found to be a result of spending more prolonged time at home [29]. Research has shown a high prevalence of mental illness during the pandemic. In some countries with a full-scale nationwide lockdown, up to 55% of the population experienced mental ill-health during the early pandemic phase [30,31,32,33]. A Swedish study found a relatively low prevalence of mental ill-health in the early phase of the pandemic, where approximately 5% worried about their mental health [17]. In the present study, adjusted behaviour was important with regard to managing the lifestyle changes necessitated by the pandemic, where families used their togetherness to strengthen relationships and find new hobbies together during the pandemic. Digital platforms were used to facilitate companionship and togetherness among those who felt loneliness or isolation. Studies have shown that digital platforms enabled connection to the outside world, social support, and engagement in activities of interest during the pandemic [22,34]. Despite an increased willingness to engage in digital platforms, participants in the present study emphasised how the elderly did not have the same opportunity to use them, because of a lack of skills, technology, or social support. A study by Peng et al. (2018), confirms that digital platforms, such as video calls and instant messaging, do not give older people the same opportunity to keep in contact at a distance compared to traditional phone calls or physical interactions [35]. The results of our study revealed that behaviours were adjusted to adapt to the restrictions of physical distancing, where work, shopping and exercises were managed from home through digital platforms; this is in line with previous studies [19,22,36,37,38,39].

In the present study, closures of swimming pools, gyms, and other activities because of the pandemic hindered the possibility of maintaining an exercise routine to relieve knee pain and manage physical function. Research has demonstrated a relationship between a sedentary lifestyle and knee pain [40] and found a relationship between knee pain and mental ill-health [41,42]. An association between a sedentary lifestyle and mental illness among Swedish citizens was found during the pandemic [17]. Individuals with knee pain may therefore be a vulnerable group during a pandemic. In the present study, outdoor activities were found to make important contributions to maintaining health and lifestyle during the pandemic among individuals with knee pain, when swimming pools, gyms and other leisure activities were closed. Research has found that Swedish citizens maintained their daily step counts to a higher degree than in other countries during the pandemic. For example, Italy showed a maximal decrease in daily steps of 49%, while Sweden had a maximal decrease of 7% [43]. The Swedish response to the pandemic, which was to avoid full-scale lockdowns, might partly explain these differences. Norwegians’ everyday life experiences showed similar results to those in the present study, where outdoor activities replaced indoor leisure activities during lockdown [31]. This may indicate that the accessibility of outdoor activities contributed to maintaining health and lifestyle during the pandemic, with or without a lockdown strategy. In previous research, adjusting behaviours by establishing a new outdoor lifestyle has been found to be associated with lower levels of mental ill-health during the pandemic, such as the experience of freely moving around outside by exercising, going for walks, hiking, being in the forest, gardening, and pursuing new hobbies; these were emphasised as easing the management of the pandemic [31,44,45,46].

In the present study, having a positive outlook on life was found to be important to maintaining health and managing the lifestyle changes caused by the COVID-19 pandemic. Valuing and cherishing relationships, being grateful for being healthy, having a job, and having the freedom to move around freely outside were considered as ways of coping with life during the pandemic. This result is in accordance with Evans et al. (2020), who found that the pandemic led to feelings of gratitude and appreciation for having a safe home, good health, time to spend together, and financial stability [29]. In the present study, the pandemic brought opportunities to reframe life, to set boundaries in order to maintain one’s health, for example, not seeing relatives or friends as often as before, or having a less stressful life, by saving time and not being obligated to drive to various leisure activities. Our results align with Sandbacken and Moss (2021), where the pandemic allowed for rethinking and reframing life. Things that had been de-prioritized for a long time could be prioritized when one lived a less stressful life and contributed to reduced negative emotions [31]. In the present study, a positive outlook was found to help cope with the situation, regardless of not receiving planned healthcare to relieve knee pain. Another study concluded that having a positive outlook on life improved health and reduced distress cause by thoughts of mortality [47]. This may illustrate how individuals with knee pain revaluated life by having a positive outlook on life to maintain health and manage lifestyle changes due to the experiences of how health and lifestyle could be affected by the COVID-19 pandemic.

In the present study, sharing responsibility by showing consideration and respect towards oneself and others was considered important in maintaining physical distance during the pandemic; for example, by doing shopping alone and when the shops were less crowded, or by supporting the elderly to maintain their health and lifestyle by buying food and medications, and leaving them outside their door. Research shows that pandemic-related changes in lifestyle were more evident among elderly living alone, where feelings of loneliness, reduction in physical activity and diminished physical functioning were more frequent than among those elderly living with others [48]. In the present study, the inability to support elderly individuals or an elderly family member created new perspectives, where life was revalued and the focus shifted from oneself to one’s loved ones. Research shows how the pandemic has brought uncertainty about the future for individuals, relatives and loved ones, which has created states of frustration, anxiety, fear, and stress [19,31]. A study found that Swedish citizens worried more about relatives’ health than their own during the pandemic [17], and it was argued in another study that worries about relatives’ and loved ones’ health motivated physical distance [30]. Another study suggested that the threat and uncertainty posed by the pandemic may push individuals toward protecting relatives and friends in their own group, rather than having solidarity with others worldwide [49].

### Methodological Discussion

In qualitative studies, trustworthiness is often explained in terms of credibility, dependability, confirmability, and transferability [25,26]. Credibility in the present study was strengthened by the transparent descriptions of data collection and data analysis and the continuous discussion between the researchers during the analysis. All participants were interviewed individually with the same interview questions in order to strengthen dependability. Not all of the co-authors have clinical experience of knee osteoarthritis, which may limit the interpretation of the results. Involving more than one researcher in the analysis encourages alternative interpretations of the results and thereby strengthens dependability. Because of the COVID-19 pandemic, the interviews were performed either at a distance, using phone or web-based videoconferencing, or face-to-face, and the variation might be a limitation. However, according to Archibald et al. [50], distance interviewing with videoconferencing services could be beneficial and even preferred. Credibility was strengthened in the present study by the researchers’ familiarity with the qualitative methodology, the careful descriptions of the data collection and analysis, and the continuous discussion between researchers during the analysis. The results were presented with appropriate quotations from different participants, representing both positive and negative experiences, which also strengthened the confirmability. A detailed description of the selection of participants and the research process were made to ensure transferability through the research. A limitation is that data were collected from only one region in Sweden, which poses a risk of selection bias. To cover variations, the participants represent a broad group of adults with different characteristics. It was considered adequate to analyse 19 interviews to detect variations and similarities among individuals with knee pain. Providing that the participants in the present study are representative of individuals with knee pain, their experience of how health and lifestyle were influenced during the pandemic could be transferable to a broader population, both nationally and internationally.

## 5. Conclusions

In conclusion, individuals with knee pain managed their health and lifestyle during the pandemic by adjusting behaviours and revaluing life. Adapting to restrictions included spending more time at home and outdoors, becoming more digital, and revaluing life by having a positive outlook on life and supporting others. However, the findings are probably similar to the general population, i.e., individuals with knee pain live similar lives as the general population despite their knee pain. The results may contribute to alternative ways of preserving health and lifestyles in different vulnerable groups under unpredictable conditions.

## Figures and Tables

**Table 1:** Participant characteristics (n = 19), presented as number (n) unless otherwise stated.

Gender, Women/Men	12/7
Age, median (range)	51 (41–62)
Civil status, Co-habiting/Living alone	16/3
Place of residence, City/countryside	11/8
Native-born/foreign-born	16/3
Level of education, Primary school/Secondary/University	2/9/6
BMI ^a^, Normal/ overweight /obesity 1/obesity 2/obesity 3	6/6/4/2/1
KOA ^b^	9
Pain group, NCP/CRP/CWP	1/10/8
Physical activity ^c^, Moderate intensity: Meeting/ not meeting recommendations Vigorous intensity: Meeting/ not meeting recommendations	10/9 4/15
Sedentary, hours median (range)	6 (1.5–11.5)

^a^ Normal, 18.5–24.9; overweight, 25.0–29.9; obesity class 1, 30.0–34.9; obesity class 2, 35.0–39.9; obesity class 3, >40, ^b^ Having a score ≥1 on the Ahlbäck scale for KOA, ^c^ WHO recommendation for moderate-intensity is 150–300 min a week; vigorous-intensity is 75–150 min a week. BMI, body mass index; KOA, radiographic knee osteoarthritis; NCP, no chronic pain; CRP, chronic regional pain; CWP, chronic widespread pain.

**Table 2:** Coding-tree.

Category	Sub-Categories	Code	Condensed Meanings Unit	Meanings Unit
Adjusting behaviours because of the COVID-19 pandemic	Becoming digital	Socialising digital	With the help of technology, have we, during the COVID-19 pandemic, been able to see each other face-to-face.	The pandemic has probably made it monotonous, but then I think, you could with the help of technology, you could… yes, see each other like this. Is this what Facetime is called? Participant no. 13.

**Table 3:** Overview of categories, and sub-categories exploring experiences of how health and lifestyle among individuals with knee pain have been influenced during the COVID-19 pandemic.

Categories	Sub-Categories
Adjusting behaviours because of the COVID-19 pandemic	Spending time at home
	Becoming digital
	Spending time outdoors
Revaluing life because of the COVID-19 pandemic	Having a positive outlook on life
	Sharing responsibility

## Data Availability

Not applicable. The data will not be shared as ethics approval for the study requires that the transcribed interviews are kept in locked files, accessible only to the researchers.
